# Influence of Zn and Sn on the Precipitation Behavior of New Al–Mg–Si Alloys

**DOI:** 10.3390/ma12162547

**Published:** 2019-08-10

**Authors:** Felix Glöckel, Peter J. Uggowitzer, Peter Felfer, Stefan Pogatscher, Heinz Werner Höppel

**Affiliations:** 1 Friedrich-Alexander-Universität Erlangen-Nürnberg, Department of Materials Science & Engineering, Institute I: General Materials Properties, 91058 Erlangen, Germany; 2 ETH Zurich, Department of Materials, Laboratory of Metal Physics and Technology, 8093 Zurich, Switzerland; 3 Chair of Nonferrous Metallurgy, Montanuniversitaet Leoben, 8700 Leoben, Austria; 4 Christian Doppler Laboratory for Advanced Aluminum Alloys, Chair of Nonferrous Metallurgy, Montanuniversitaet Leoben, 8700 Leoben, Austria

**Keywords:** Al–Mg–Si alloy, Zn, Sn, paint bake, Al–Mg–Si–Zn–Sn alloys, cluster formation, hardening behavior

## Abstract

In this study, we demonstrate how Zn and Sn influence hardening behavior and cluster formation during pre-aging and paint bake treatment in Al–Mg–Si alloys via hardness tests, tensile tests, and atom probe tomography. Compared to the standard alloy, the Sn-modified variant shows reduced cluster size and yield strength in the pre-aged condition. During the paint bake cycle, the clusters start to grow very fast and the alloy exhibits the highest strength increment. This behavior is attributed to the high vacancy binding energy of Sn. Adding Zn increases the formation kinetics and the size of Mg–Si co-clusters, generating higher yield strength values for both the pre-aged and paint baked conditions. Simultaneous addition of Zn and Sn creates a synergistic effect and produces an alloy that exhibits moderate strength (and good formability) in the pre-aged condition and accelerated hardening behavior during the paint bake cycle.

## 1. Introduction

Al–Mg–Si alloys (6xxx series) are characterized by medium to high strength, high fracture toughness and good resistance against corrosion and stress corrosion cracking. They also feature good weldability [1,2]. For these reasons, Al–Mg–Si alloys are widely used for components in various applications. Due to their good recyclability and the excellent formability of sheet materials in the T4 condition, they are particularly attractive for the automotive industry [3,4]. Modern automotive sheet alloys are designed to harden during the so-called paint bake cycle (PBC), where the complete car body is heated to elevated temperatures for a limited period. Both a pronounced increase in strength during the PBC and a reduced tendency for natural aging (NA) prior to the PBC must be ensured. Recently, an attempt was made to reduce the paint bake temperature in order to realize polymer-based components and save energy.

Diffusion processes and the resulting cluster formation directly after quenching are known to cause natural aging in Al–Mg–Si alloys. This produces an undesirable increase in strength, a decrease in formability, and a reduction in age hardening potential [5,6,7]. To counteract these effects, a pre-aging heat treatment procedure is usually performed. This is typically carried out immediately after quenching at temperatures between 60 and 200 °C [8]. According to Zhen and Kang [9], pre-aging is assumed to suppress negative cluster formation during natural aging, but generates a high number density of β′′ phase nuclei, stimulating the subsequent paint bake response. An alternative method for suppressing the natural aging effect was recently presented by Pogatscher et al. [10,11,12]. Their studies showed that adding Sn inhibits cluster formation and improves subsequent artificial aging. This effect is attributed to the trapping of vacancies by Sn atoms at low temperatures. At elevated temperatures, i.e., during the PBC, Sn releases the vacancies and fast non-equilibrium diffusion is enabled. This desirable Sn addition effect, called “diffusion on demand” [10], has been confirmed by other authors [13,14,15,16].

While it is desirable to retard the kinetics of cluster formation at room temperature, the reverse is true for artificial aging. For paint bake hardening, using Zn additions in Al–Mg–Si alloys has proven to be an effective approach [17,18,19,20]. Saito et al. [21] showed that hardness increases after ageing at 185 °C if 1 wt % Zn is added to an Al–Mg–Si alloy. They detected an increased number density of needle-shaped precipitates from the Al–Mg–Si system, but no phases containing zinc. Guo et al. [18] carried out DSC measurements on Al–Mg–Si alloys with varying Zn content. They observed a shift of the precipitation peak at 250 °C, known as the β′′-peak, to lower temperatures when 3 wt % Zn was added. They also reported that the type of precipitate is not affected by Zn addition, i.e., no Mg–Zn precipitates were observed [18].

In this study, the above approaches of adding Sn to retard natural aging and alloying Zn to promote aging in the paint PBC were combined in various experimental alloys to investigate hardening behavior and cluster formation during pre-aging and PBC. Four different experimental alloys were investigated: (1) standard Al–Mg–Si as reference; (2) Al–Mg–Si with Zn addition; (3) Al–Mg–Si with Sn addition; and (4) Al–Mg–Si with both Zn and Sn addition. Via hardness and atom probe tomography (APT) measurements, we characterized and evaluated the influence of the alloying elements individually and in combination.

## 2. Materials and Methods 

The alloys were produced by gravity casting at AMAG Rolling GmbH, Ranshofen, Austria. The Al–Mg–Si base alloy with different additions of other elements were melted in an induction furnace and then cast into preheated gravity dies to obtain ingots with a size of 170 × 80 × 40 mm^3^. Subsequently, the ingots were homogenized in a two-step procedure at temperatures of 540 and 565 °C. Afterwards they were first hot- and then cold-rolled to 1.5 mm thick sheets. The solution heat treatment took place in an air furnace at 560 °C for 15 min, and was followed by water quenching. Subsequently, the sheets were pre-aged at a temperature of 110 °C for 5 h (denoted as the “T4P” condition). Afterwards, 2% of pre-straining was applied. Half of the samples were tested in this condition and the other half was subjected to a modified paint bake hardening treatment/cycle (PBC) at a reduced temperature of 165 °C for 20 min (“T6P” condition). The alloy with Zn and Sn additions was also overaged to the “T7” condition at 170 °C for 120 h.

The nominal compositions of the experimental alloys are listed in Table 1. It should be noted that all alloys exhibit the same Mg and Si levels and differ only in their Zn and Sn content.

To analyze the hardening behavior during pre-aging and PBC, Brinell hardness measurements were carried out on a Diatestor 2 RC (Wolpert, Aachen, Germany). A ball diameter of 2.5 mm, a load of 306 N (31.25 kp) and a dwell time of 10 s were applied according to DIN EN ISO 6506-1. Tensile tests were also performed on an Inspekt 250 (Hegewald & Peschke, Nossen, Germany) machine according to EN ISO 6892-1 to analyze mechanical properties.

To analyze the clustering behavior at the atomic scale, APT measurements were conducted. For this purpose, small 15 × 15 mm² samples were ground to a thickness of 0.5 mm. Then, small rods of 15 × 15 × 0.5 mm^3^ were cut from these sheets and electropolished using a standard two-step method to form a small tip. First, we performed “rough polishing” with an electrolyte consisting of 25% perchloric in 75% acetic acid. Then, we carried out “fine polishing” using a solution of 2% perchloric acid in butanol. The measurements were conducted on a LEAPTM 4000X HR (Cameca, Madison, WI, USA) in voltage mode at a temperature of 40 K. This system has a detection efficiency of ~37%, meaning only 37% of the atoms arriving at the detector are amplified. We used a pulse fraction of 20% and a detection rate of 1%. Data reconstruction was carried out using IVASTM 3.6.8 software from Cameca. To minimize influences from crystallographic artefacts, we limited our analysis to cubes with an identical volume of 30 × 30 × 100 nm^3^ which lay away from major crystallographic poles. The clustering of the atoms of interest was determined by an algorithm based on Voronoi tessellation of the solute atoms and Delaunay triangulation to test the random distribution of the solid solution (as described elsewhere [22,23,24]). The data were analyzed using custom scripts in MATLAB R2016a from MathWorks. 3D atom maps, cluster size, Mg/Mg + Si distribution, and proxigrams were used to visualize the results of the APT in this study. The 3D atom maps show a 3D picture of the most important atoms: Si, Mg, Zn, and Sn. The Mg/Mg + Si distribution is deployed to display detailed information about the Mg and Si ratio within the clusters. The proxigrams illustrate the element content in the cluster, starting in the center of a cluster. For this analysis, the data of all identical cluster types are averaged, and the clusters are regarded as spheres. Of course, this procedure does not account for the exact geometry of the clusters, but it reflects for the differences between the various conditions and alloys. The mean cluster size of the Mg–Si co-clusters indicates how many Mg and Si atoms participate in the local elemental enrichment.

It is important to note that for reasons of simplification and ease of readability, all detected aggregates were referred to as clusters according to the cluster detection algorithm used, even if different types of precursor of the β phase might be present.

## 3. Results

### 3.1. Hardness and Tensile Tests

**Figure 1:**
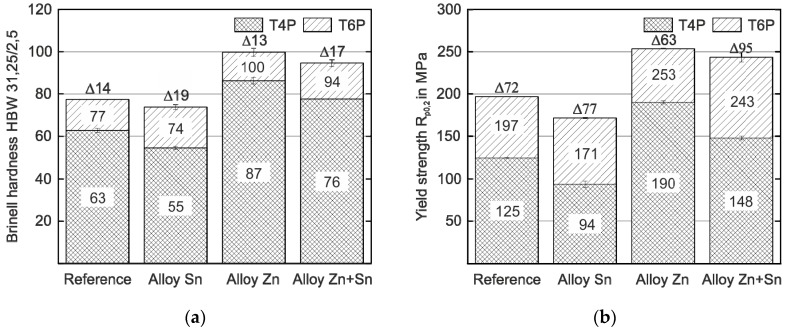
Hardness (**a**) and yield strength values (**b**) of the reference and experimental alloys in the T4P and T6P conditions (average scatter ±1 HBW and ±2 MPa, respectively). The hardness and strength difference, ∆, between T4P and T6P states is indicated.

Figure 1 shows the hardness and yield strength values of the experimental alloys in the T4P and T6P conditions. Significant differences in hardness and yield strength are obtained depending on the type of alloy already in the T4P condition. Compared to the reference alloy without Sn or Zn, the strength of the alloy with Sn addition is slightly lower, while the alloy with Zn addition exhibits the highest strength value. When Zn and Sn are added simultaneously (Alloy Zn + Sn), hardness and yield strength are significantly higher than the reference value and only slightly lower compared to Alloy Zn. After the paint bake treatment (T6P condition), the various hardening potentials of the alloys become discernible. Alloy Sn has the greatest hardness increase (19 HBW) and almost reaches the T6P strength of the Reference. For Alloy Zn and Alloy Zn + Sn, the hardness and yield strength in the T6P condition is higher compared to the Reference and both alloys exhibit comparable strength levels after bake hardening. Obviously, the paint bake response of Alloy Zn + Sn is significantly higher than that of Alloy Zn (17 HBW compared to 13 HBW and 95 MPa compared to 63 MPa). This is highly desirable because it enables forming in a state of low strength and high ductility, while still generating high final hardness and yield strength.

### 3.2. Atom Probe Tomography

To shed light on the origins of the age hardening responses, we carried out an extensive APT analysis. This should make it possible to correlate the mechanical properties with the presence of the atomic clusters responsible for hardness evolution.

#### 3.2.1. Comparison of T4P and T6P Conditions

##### Reference Alloy

**Figure 2:**
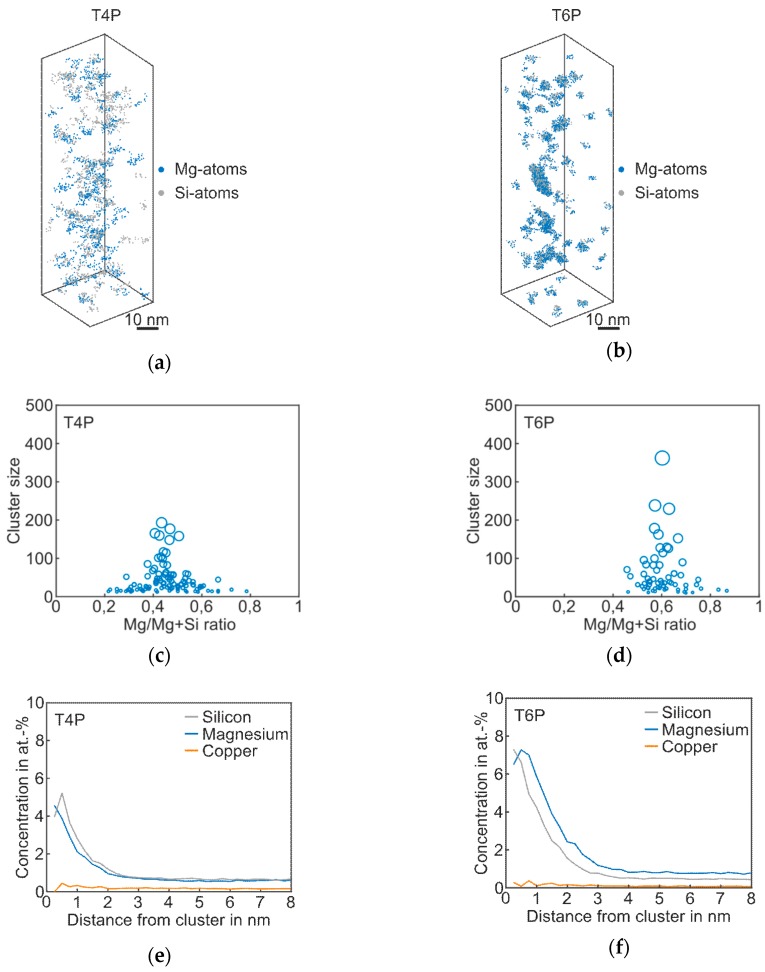
APT analyses of Reference alloy visualizing the 3D atom maps of Mg–Si co-clusters (**a**,**b**) in T4P and T6P conditions. The corresponding cluster size distributions and proxigrams are shown in (**c**–**f**). The different circle diameters in (**c**) and (**d**) indicate the cluster size.

Figure 2 shows the results of atom probe tomography measurements of the *Reference* in T4P and T6P conditions, and Table 2 displays the corresponding cluster properties. In Figure 2a, small aggregations of Mg and Si atoms are visible in the atom maps; they indicate clustering. Figure 2b presents the T6P condition after paint bake treatment. Significant cluster growth is recognizable. This can also be seen in Figure 2c,d: the larger circles indicate bigger clusters in T6P. Small sporadic needle shaped phases also occur in this condition. It is also worth mentioning that the Mg/Mg + Si ratio changes from 0.4 in T4P condition to 0.6 in T6P condition, i.e., the clusters contain more Mg than Si after the paint bake treatment (see average Mg/Si ratio in Table 2). This assumption is confirmed by the proxigrams in Figure 2e,f. The latter show, starting from the center of the clusters, the concentration trends of the most important phase forming elements Mg, Si, and Cu. The Mg and Si concentrations are increased to a distance of ~2 nm in T4P and ~3 nm in T6P. Afterwards, at a greater distance, they show a constant level, which indicates the matrix concentration. There are no big differences in the Cu concentration trend, which indicates that Cu participates only slightly in the cluster. Table 2 presents data for the mean cluster size, average Mg/Si ratio, and number density of Mg–Si clusters in T4P and T6P conditions. It is obvious that the mean cluster size doubles and the number density decreases after PBC. These values are reflected well in the 3D atom maps in Figure 2a,b.

##### Alloy Sn

**Figure 3:**
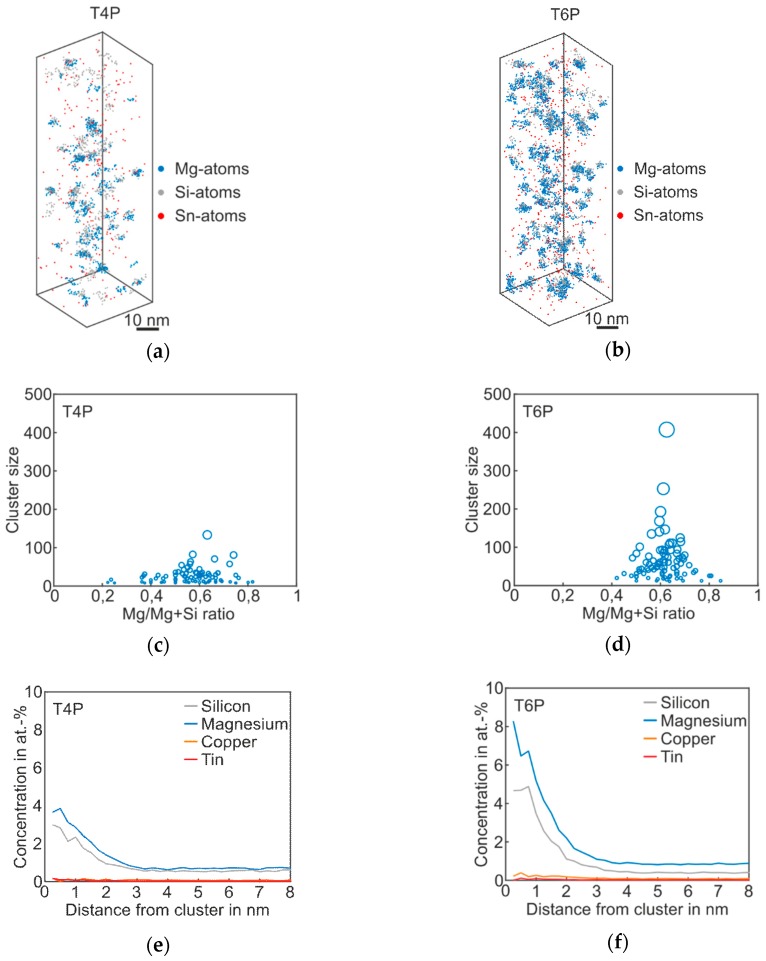
APT analyses of Alloy Sn visualizing the 3D atom maps of Mg–Si co-clusters (**a**,**b**) in T4P and T6P conditions. The corresponding cluster size distributions and proxigrams are shown in (**c**–**f**). The different circle diameters in (**c**) and (**d**) indicate the cluster size.

Figure 3 shows the atom probe results of *Alloy Sn* in the T4P and T6P states, and Table 3 the corresponding cluster properties in the same fashion as for the *Reference*. The 3D atom map in Figure 3a exhibits only very small cluster agglomerations. It follows that Sn does not participate in clustering, but, instead, is distributed homogenously in the matrix. However, for the T4P condition, adding Sn produces a significantly reduced mean cluster size. In condition T6P, however, the mean cluster size is much larger, increasing from 24 to 63 (Table 3). Sn atoms in condition T6P are also not enriched in the clusters. Interestingly, the number density does not change between the two conditions. The cluster size distributions in Figure 3c,d display the difference between the cluster evolutions of T4P and T6P. It is noticeable that the Mg/Mg + Si ratio does not change after the paint bake treatment and remains at ~0.6. Thus, the clusters in T4P condition are Mg-rich and not Si-rich as in the Reference alloy. This is also visible in the proxigrams in Figure 3e,f, which show higher Mg cluster content in both conditions. The Cu and Sn concentrations remain unaffected.

##### Alloy Zn

**Figure 4:**
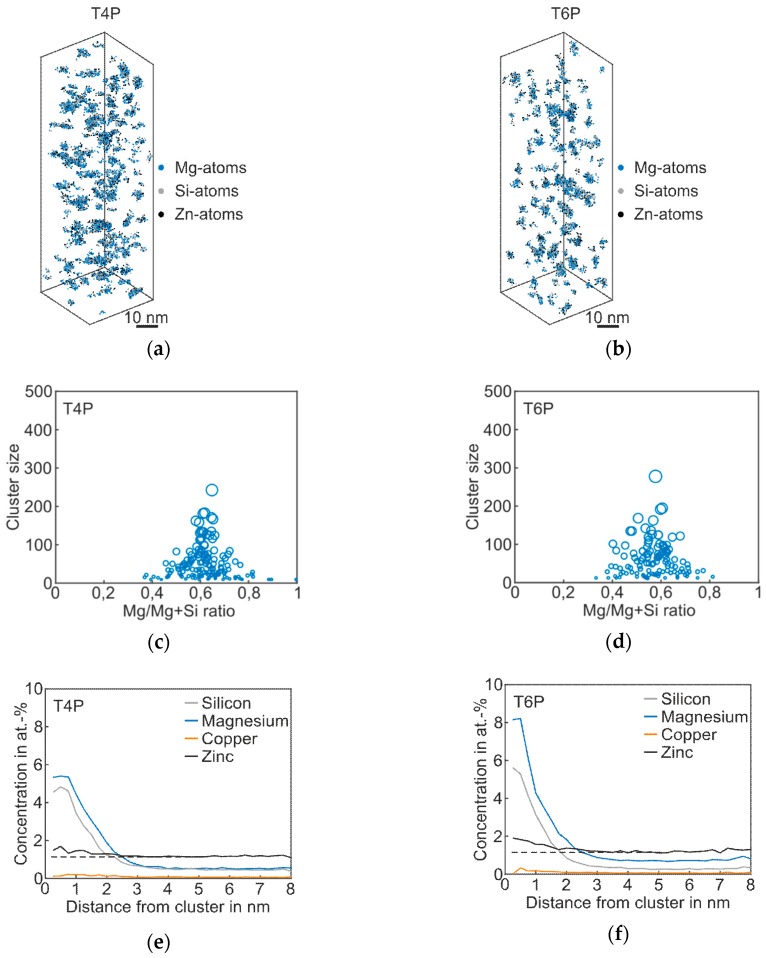
APT analyses of Alloy Zn visualizing the 3D atom maps of Mg–Si co-clusters (**a**,**b**) in T4P and T6P conditions. The corresponding cluster size distributions and proxigrams are shown in (**c**–**f**). The different circle diameters in (**c**) and (**d**) indicate the cluster size. The dotted lines in (**e**,**f**) reflect the matrix concentration level of Zn.

The results of the APT analysis of *Alloy Zn* are presented in Figure 4, and the corresponding cluster properties in Table 4. Compared to the T4P condition of the *Reference*, the Mg–Si co-clusters in the 3D atom maps appear bigger, which is also confirmed by the mean cluster size of 49; see Table 4. As shown in the proxigram in Figure 4e, the Zn atoms partially agglomerate in the Mg–Si co-clusters. The Zn concentration increases when compared to the matrix concentration, which is visualized by the dotted line. After paint bake treatment, the clusters are somewhat coarser, but the overall structure does not change strongly, as shown in Figure 4b,d. The clusters still consist primarily of Mg and Si atoms, with a slight enrichment in Zn. Typical Zn-containing phases such as η’ were not detected [25]. Thus, we still assign this type of alloy to the Mg–Si–family, despite the elevated Zn content. The Mg/Mg + Si ratio does not change during paint bake treatment, but remains at a value of ~0.6, i.e., the average Mg/Si ratio remains at 1.7 (Table 4). Compared to the *Reference*, the number density in both T4P and T6P conditions is higher.

##### Alloy Zn + Sn

**Figure 5:**
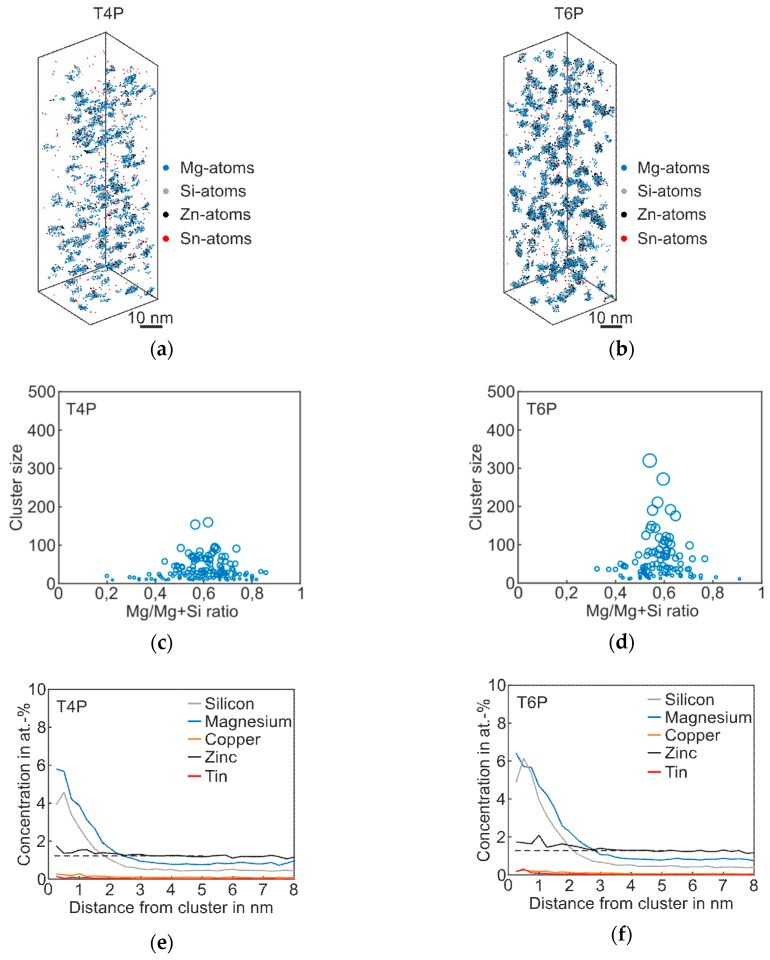
APT analyses of Alloy Zn + Sn visualizing the 3D atom maps of Mg–Si co-clusters (**a**,**b**) in T4P and T6P conditions. The corresponding cluster size distributions and proxigrams are shown in (**c**–**f**). The different circle diameters in (**c**) and (**d**) indicate the cluster size. The dotted lines in (**e**,**f**) reflect the matrix concentration level of Zn.

The APT results of the combination of Zn and Sn in *Alloy Zn + Sn* and the corresponding cluster properties are presented in Figure 5 and Table 5. 

The co-clusters of Mg and Si in Figure 5a appear to be not as numerous and large as those of *Alloy Zn*. They are smaller in size but not as small as those of *Alloy Sn*. Much bigger clusters are visible after paint bake treatment (Figure 5b). This is also evident in the mean cluster size (Table 5). As in *Alloy Sn* and *Alloy Zn*, the Mg-enrichment is more pronounced than that of Si (Mg/(Mg + Si) ≈ 0.6) and does not change during the PBC. Zn is slightly enriched in the clusters.

#### 3.2.2. T7 Condition

##### Alloy Zn + Sn

**Figure 6:**
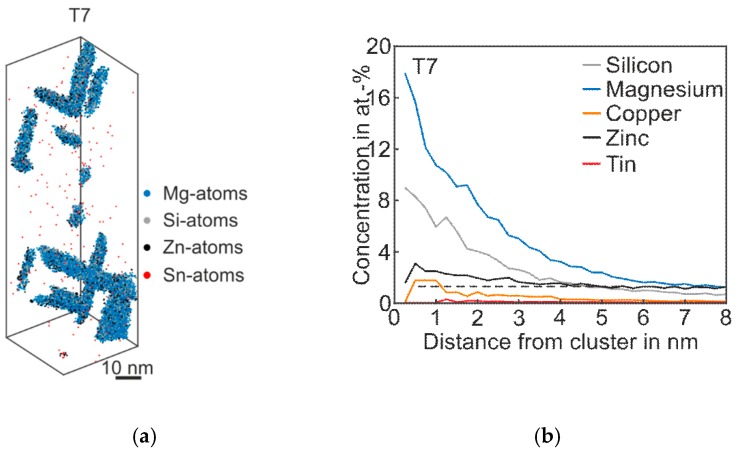
APT analyses of Alloy Zn + Sn visualizing the 3D atom maps of Mg–Si co-clusters (**a**) in T7 condition. The corresponding proxigram is presented in (**b**). The dotted line indicates the matrix concentration level of Zn.

Figure 6 shows the 3D atom map and the corresponding proxigram of *Alloy Zn + Sn* in the overaged T7 condition. As expected, coarse plate- and needle-like phases are visible. The proxigram in Figure 6b illustrates the Mg content, which is two times higher than Si. The Zn concentration within the phases is twice as high as the matrix concentration, and the Cu content also increases in this condition. Interestingly, even after long-term artificial aging, there is still no η-phase (MgZn_2_) and no precursors are detectable [26].

## 4. Discussion

**Figure 7:**
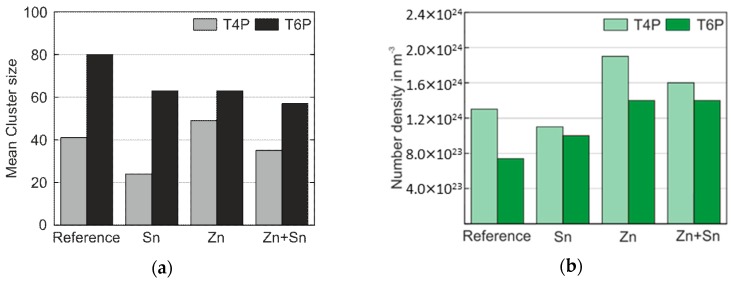
(**a**) Mean cluster size of the Mg–Si co-clusters in the T4P and T6P conditions; (**b**) the corresponding number density, N.

The presented results provide a fairly clear picture of how the modifying elements Sn, Zn, and Sn + Zn influence the early stages of precipitate formation and, thus, the mechanical properties as represented by hardness and yield strength values of Al–Mg–Si alloys. A summary of the most relevant results of the ATP cluster analysis is presented in Figure 7. For both the T4P and T6P conditions, Figure 7a shows the mean cluster size of the Mg–Si co-clusters, and Figure 7b displays the number density of clusters in these states. Sn obviously retards cluster growth in condition T4P but accelerates it during PBC. On the other hand, the addition of Zn generates an increased number density of clusters independent of the presence of Sn.

**Figure 8:**
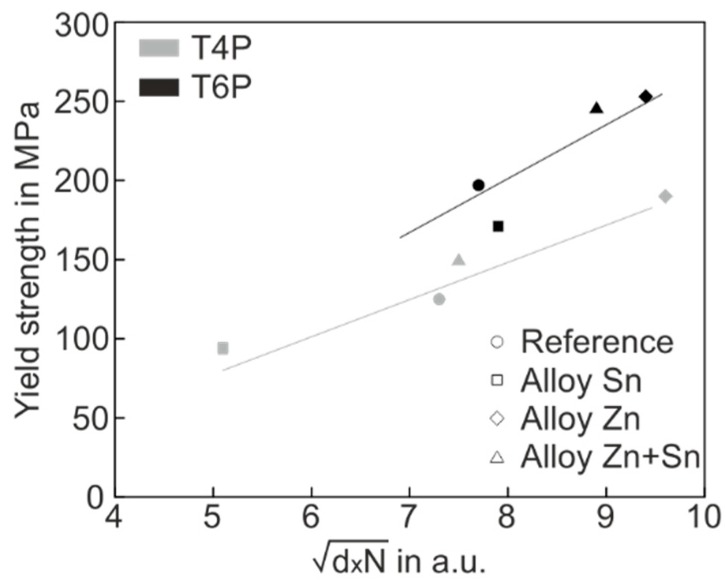
Yield strength versus $\sqrt{d*N}$ of all experimental alloys in the T4P (grey) und T6P (black) conditions.

Before discussing the elemental influences on cluster size and cluster number density, we will take a closer look at the relationship between cluster formation and yield strength. Hardening is controlled by interactions between dislocations and clusters. An appropriate approach to describing the flow stress increase by the presence of clusters delivers the “dispersed barrier model” [27,28],

(1) $$\Delta \sigma =\alpha M\mu b\sqrt{dN},$$

where *M* is the Taylor factor, *µ* the shear modulus, *b* the Burgers vector, *d* and *N* the cluster obstacle size and cluster number density, and α the cluster barrier strength. We now take the liberty of simplification and set the mean cluster size determined by APT as obstacle size d. For the various alloys in conditions T4P and T6P, we calculate the $\sqrt{d*N}$ values listed in Table 6 and illustrate the correlation to the yield strength values in Figure 8.

In Figure 8, two special features are easy to recognize. Firstly, the yield stress values of both T4P and T6P states correlate quite well with $\sqrt{d*N}$, which supports the applicability of the hardening model. Secondly, the T6P data (black) lie predominantly above the T4P data (grey), indicating a greater hardening effect on the part of the T6P clusters, i.e., the cluster barrier strength α in Equation (1) is significantly higher for T6P clusters than for T4P clusters. The greater barrier strength of the T6P clusters is not surprising because these clusters were transformed into a more mature state by the PBC treatment.

We now discuss the influence of Sn, Zn, and Sn + Zn on the cluster parameters.

*Alloy Sn* shows a reduced number density of Mg–Si co-clusters and a smaller mean cluster size compared to the Reference. We attribute these effects to the vacancy binding effect of Sn, as often described in the literature [10,11,12,29]. At the T4P temperature, substitutional diffusion is inhibited. At the PBC temperature of 165 °C, however, Sn releases the trapped vacancies, generating rapid cluster growth.

With the addition of Zn in *Alloy Zn*, there is an increase in both mean cluster size and especially number density. Zinc does not form clusters by itself, but stimulates the nucleation and growth of Mg–Si co-clusters. These findings agree well with those in the literature [17,30,31,32]. Zn has an attractive vacancy binding energy [33,34]) and it can thus be assumed that when Zn atoms are present, more vacancies survive the quenching procedure. In contrast to Sn addition, however, the critical temperature for releasing the trapped vacancies is already exceeded during the T4P treatment at 110 °C, and rapid nucleation and growth can take place. Only a slight enrichment of Zn in the clusters was detected, which might be related to the Mg–Zn formation enthalpy (−6.1 kJ/mol), calculated by Wolverton [35]. Guo et al. [31] postulate that the Mg atoms can diffuse more easily to the clusters when Zn is present, perhaps explaining not only the faster nucleation and growth of the clusters but also the increased concentration of Mg atoms within the clusters (mean Mg/Si ratio ≈ 1.7). In accordance with our findings, Yuan et al. [30] also detected no Mg–Zn phases in Al–Mg–Si–Cu–Zn–Mn. They also observed an enhanced nucleation process and a higher number density of β′′ precipitates. Ding et al. [17] measured zinc containing phases after excessive artificial aging (170 °C for 120 h or 200 °C for 24 h). In our study, however, no η-phase or their precursors were detected, not even in overaged T7 condition after long-term artificial aging (Figure 6).

*Alloy Zn + Sn* shows a synergetic effect of Zn and Sn. The obtained number density and mean cluster size of the Mg–Si co-clusters are reduced compared with those of *Alloy Zn* in T4P. Here, too, adding Sn also retards the formation of clusters. However, the mean cluster size and the number density have higher values than those of *Alloy Sn* due to the abovementioned contribution of Zn to the nucleation and growth of clusters.

## 5. Conclusions

This study investigated the influence of Zn and Sn on hardening behavior and cluster formation in Al–Mg–Si alloys. The following conclusions were drawn:

- Adding Sn produces a decrease in hardness in the pre-aged condition because Sn-trapping of vacancies suppresses cluster formation. The vacancies are released from the Sn atoms during the paint bake process, leading to rapid growth of already existing clusters. Consequently, the hardness increase from the pre-aged to the paint baked condition is more pronounced than in the Sn-free reference alloy.
- Adding Zn promotes Mg–Si co-cluster formation. The number density and average size of the Mg–Si co-clusters are significantly increased, generating the highest hardness even in the pre-aged condition. During the paint bake cycle, further cluster growth takes place and, thus, the Zn-modified alloy shows the highest hardness value.
- Simultaneous addition of both Sn and Zn produces a synergetic effect. The size of the clusters is slightly decreased by the presence of Sn, but their number density stays high because of Zn. This causes a hardness which is lower than that of the solely Zn-modified alloy in the pre-aged condition, but more pronounced hardening is achieved during the paint bake cycle. This is preferred, since it enables the forming of sheets in the soft pre-aged condition and application in the hard paint baked state.

In general, the results show that the combined addition of Sn and Zn to Al–Mg–Si alloys is of particular interest when considering the applicability of reduced paint bake temperatures.

## Figures and Tables

**Table 1:** Composition of experimental alloys in wt %.

Alloy	Al	Mg	Si	Zn	Sn	Cu
Reference	Bal.	1.0	0.5	-	-	0.17
Alloy Sn	Bal.	1.0	0.5	-	0.04	0.17
Alloy Zn	Bal.	1.0	0.5	3.0	-	0.17
Alloy Zn + Sn	Bal.	1.0	0.5	3.0	0.04	0.17

**Table 2:** Properties of the Mg–Si clusters of Reference alloy in T4P and T6P conditions.

Condition	Mean Cluster Size	Average Mg/Si Ratio	Number Density
T4P	41	0.8	1.3 × 10^24^ m^−3^
T6P	80	1.5	7.4 × 10^23^ m^−3^

**Table 3:** Properties of the Mg–Si clusters of Alloy Sn in T4P and T6P conditions.

Condition	Mean Cluster Size	Average Mg/Si Ratio	Number Density
T4P	24	1.4	1.1 × 10^24^ m^−3^
T6P	63	1.6	1.0 × 10^24^ m^−3^

**Table 4:** Properties of the Mg–Si clusters of Alloy Zn in T4P and T6P conditions.

Condition	Mean Cluster Size	Average Mg/Si Ratio	Number Density
T4P	49	1.7	1.9 × 10^24^ m^−3^
T6P	63	1.7	1.4 × 10^24^ m^−3^

**Table 5:** Properties of the Mg–Si clusters of Alloy Zn + Sn in the T4P and T6P condition.

Condition	Mean Cluster Size	Average Mg/Si Ratio	Number Density
T4P	35	1.5	1.6 × 10^24^ m^−3^
T6P	57	1.5	1.4 × 10^24^ m^−3^

**Table 6:** Hardening variables $\sqrt{d*N}$ of the Mg–Si co-clusters in T4P and T6P condition (data in arbitrary units (a.u.)).

Alloys	$\sqrt{d*N}\;\mathrm{in}\;a.\;u.$	
	T4P	T6P
Reference	7.3	7.7
Alloy Sn	5.1	7.9
Alloy Zn	9.6	9.4
Alloy Zn + Sn	7.5	8.9
